# Supplementary material for: Photosynthesis Performance at Different Growth Stages, Growth, and Yield of Rice in Saline Fields
Source: Plants (Basel). 2023 May 7;12(9):1903. doi: 10.3390/plants12091903 (PMC10181347; doi:10.3390/plants12091903)
Supplement: Supplementary file 1 [file plants-12-01903-s001.zip › plants-2314580-supplementary.pdf]

Supplementary Table S1. Environmental conditions near the rice fields including monthly total rainfall, rainy day number, mean relative humidity (RH), minimum temperature (T_{min}) and maximum temperature (T_{max}) during January to December 2021 recorded by Upper Northeastern Meteorological Center, Mueang Khon Kaen District, Khon Kaen, Thailand. The electrical conductivity (EC) of soil and water of the rice fields including non-saline, semi-saline and heavy saline were measured during July to Nov 2021 (gray area).

Month	Total of rainfall (mm)	Rainy day number (day)	RH (%)	T_{min} (°C)	T_{max} (°C)	Electrical conductivity (dS m^{-1}) in the fields					
						Soil			Water		
						Non-saline	Semi-saline	Heavy-saline	Non-saline	Semi-saline	Heavy-saline
January	0.0	0	58.64 ± 4.2	15.81 ± 3.0	28.86 ± 3.0	—	—	—	—	—	—
February	21.4	2	58.46 ± 8.5	19.01 ± 1.6	31.92 ± 3.3	—	—	—	—	—	—
March	92.3	6	66.48 ± 7.6	23.73 ± 1.9	34.61 ± 2.9	0.86 ± 0.11	13.45 ± 5.8	45.27 ± 13.07	—	—	—
April	135.8	9	71.06 ± 9.1	24.87 ± 1.3	34.57 ± 3.2	—	—	—	—	—	—
May	75.9	11	72.58 ± 7.0	25.69 ± 1.1	35.52 ± 2.0	0.12 ± 0.01	2.87 ± 0.02	2.53 ± 0.01	—	—	—
June	142.3	13	74.06 ± 9.1	25.47 ± 1.2	34.48 ± 2.9	—	—	—	—	—	—
July	114.8	15	78.16 ± 8.4	25.08 ± 1.0	33.40 ± 2.4	0.06 ± 0.01	1.08 ± 0.09	1.12 ± 0.01	0.06 ± 0.01	2.26 ± 0.22	2.97 ± 0.11
August	141.7	17	78.19 ± 7.1	25.04 ± 1.0	33.80 ± 1.8	0.04 ± 0.01	1.21 ± 0.14	1.32 ± 0.40	0.21 ± 0.25	1.78 ± 0.07	2.07 ± 0.05
September	271.5	23	86.83 ± 4.5	24.21 ± 0.8	31.58 ± 1.7	0.04 ± 0.03	1.05 ± 0.16	1.33 ± 0.29	0.06 ± 0.01	1.31 ± 0.28	1.48 ± 0.17
October	136.7	14	81.22 ± 5.5	23.44 ± 1.6	31.30 ± 2.2	0.48 ± 0.31	2.86 ± 1.58	4.11 ± 1.95	dry	dry	dry
November	0.0	0	69.93 ± 5.4	21.40 ± 2.0	31.37 ± 1.6	0.87 ± 0.14	4.05 ± 1.22	4.47 ± 1.51	dry	dry	dry
December	0.0	0	60.41 ± 5.0	17.78 ± 2.0	29.40 ± 1.5	—	—	—	—	—	—

Supplementary Table S2. SPAD values and photosynthetic parameters including net photosynthesis (P_n), stomatal conductance (gs), transpiration rate (T_r), water use efficiency (WUE), effective quantum yield of PSII photochemistry (ΦPSII), maximum quantum yield efficiency of PSII ($F_{v'}/F_{m'}$) and electron transport rate (ETR) of five rice genotypes including Pokkali, RD73, CSSL8-94, TSKC1-144 and KDML105 growing under non-saline, semi-saline and heavy-saline field. The data were measured at six rice growth stages including early vegetative, stem elongation, early booting, flowering, milky and mature. Means which are significantly different ($p < 0.05$ and $p < 0.01$) among genotypes for each saline field and field across genotypes are denoted by different lower-case letters. For each genotype means which are significantly different ($p < 0.05$) among growth stage are denoted with capital letters. Data shows mean of four replicates ± standard error (SE).

Parameter	Saline field	Rice genotype	Early vegetative	Stem elongation	Early booting	Flowering	Milky	Mature	[2] Critical-*p* value
SPAD value	Non-saline	Pokkali	31.40 ± 0.60 bB	36.42 ± 0.33 bA	36.90 ± 0.48 A	36.55 ± 0.65 A	36.30 ± 0.80 bA	27.55 ± 1.27 bC	$p < 0.01$
		RD73	34.47 ± 0.61 aDE	38.45 ± 0.57 aBC	36.32 ± 0.85 CD	41.00 ± 1.75 AB	42.20 ± 0.75 aA	32.30 ± 0.90 aE	$p < 0.01$
		CSSL8-94	34.47 ± 0.40 aC	37.25 ± 0.29 abB	36.02 ± 0.39 BC	40.77 ± 1.34 A	41.35 ± 1.01 aA	30.52 ± 0.26 abD	$p < 0.01$
		TSKC1-144	33.30 ± 0.33 abB	38.07 ± 0.41 aA	34.72 ± 1.12 B	40.40 ± 1.11 A	33.80 ± 1.06 cB	28.45 ± 0.65 bC	$p < 0.01$
		KDML105	34.22 ± 0.58 aCD	37.47 ± 0.42 abB	35.07 ± 0.60 C	39.76 ± 0.25 A	41.56 ± 0.64 aA	32.75 ± 1.25 aD	$p < 0.01$
		[1] Critical-*p* value	$p < 0.01$	$p < 0.05$	ns	ns	$p < 0.01$	$p < 0.01$	
		**mean**	**33.57 ± 0.34 cD**	**37.53 ± 0.23 bB**	**35.81 ± 0.35 bC**	**39.69 ± 0.59 bA**	**39.04 ± 0.85 bA**	**30.31 ± 0.60 cE**	**$p < 0.01$**
	Semi-saline	Pokkali	43.80 ± 1.13 A	42.22 ± 1.05 AB	39.92 ± 0.76 bB	42.30 ± 0.76 bcAB	43.45 ± 1.45 A	33.22 ± 2.32 C	$p < 0.01$
		RD73	43.57 ± 0.90	40.50 ± 2.41	46.62 ± 1.90 a	48.65 ± 1.83 a	48.42 ± 1.89	43.37 ± 3.11	ns
		CSSL8-94	43.10 ± 1.16 A	37.70 ± 1.48 C	42.60 ± 1.69 abAB	41.60 ± 0.97 cABC	45.30 ± 1.07 A	38.85 ± 2.18 BC	$p < 0.05$
		TSKC1-144	45.82 ± 1.69	39.75 ± 0.85	40.22 ± 1.87 b	42.65 ± 1.96 bc	43.37 ± 1.65	37.75 ± 2.37	ns
		KDML105	44.20 ± 0.74 A	39.27 ± 1.80 B	46.27 ± 1.01 aA	46.87 ± 1.19 abA	47.42 ± 1.18 A	37.05 ± 3.06 B	$p < 0.01$

Parameter	Saline field	Rice genotype	Early vegetative	Stem elongation	Early booting	Flowering	Milky	Mature	[2] Critical-*p* value
		[1] Critical-*p* value	ns	ns	$p < 0.05$	$p < 0.05$	ns	ns	
		**mean**	**44.10 ± 0.51 aAB**	**39.89 ± 0.73 aC**	**43.13 ± 0.89 aB**	**44.41 ± 0.86 aAB**	**45.59 ± 0.75 aA**	**38.05 ± 1.29 aC**	**$p < 0.01$**
	Heavy-saline	Pokkali	40.85 ± 1.55 B	46.46 ± 1.28 aA	39.87 ± 1.24 B	40.52 ± 1.04 bB	47.52 ± 0.35 aA	28.40 ± 1.23 bC	$p < 0.01$
		RD73	41.60 ± 1.27 B	40.57 ± 1.04 bB	44.02 ± 1.95AB	45.45 ± 0.20 aA	47.20 ± 2.31 aA	33.60 ± 1.91 abC	$p < 0.01$
		CSSL8-94	42.65 ± 0.56 AB	37.87 ± 1.72 bC	41.65 ± 1.51 ABC	44.67 ± 1.61 aA	43.72 ± 0.63 abA	38.92 ± 1.91 aBC	$p < 0.05$
		TSKC1-144	41.42 ± 1.47 AB	37.42 ± 0.41 bBC	43.35 ± 2.28 A	39.30 ± 1.56 bAB	40.50 ± 1.40 bAB	33.07 ± 2.89 bC	$p < 0.05$
		KDML105	41.80 ± 0.88 B	40.90 ± 0.73 bB	43.50 ± 0.83 AB	46.02 ± 1.07 aA	45.45 ± 0.66 aA	32.10 ± 1.86 bC	$p < 0.01$
		[1] Critical-*p* value	ns	$p < 0.01$	ns	$p < 0.01$	$p < 0.01$	$p < 0.05$	
		**mean**	**41.66 ± 0.50 bBC**	**40.64 ± 0.87 aC**	**42.48 ± 0.74 aBC**	**43.19 ± 0.79 aAB**	**44.88 ± 0.78 aA**	**33.22 ± 1.12 bD**	**$p < 0.01$**
	**[3] Critical-*p* value**		**$p < 0.01$**	**$p < 0.01$**	**$p < 0.01$**	**$p < 0.01$**	**$p < 0.01$**	**$p < 0.01$**	
P_n	Non-saline	Pokkali	23.13 ± 0.64 A	23.84 ± 0.79 A	22.26 ± 1.41 bA	19.40 ± 0.55 bB	18.29 ± 0.43 cB	13.33 ± 0.54 C	$p < 0.01$
		RD73	23.29 ± 1.25 B	25.58 ± 1.22 AB	27.98 ± 1.07 aA	26.58 ± 0.82 aA	25.63 ± 0.54 aAB	13.90 ± 1.09 C	$p < 0.01$
		CSSL8-94	24.21 ± 0.38 A	24.44 ± 1.56 A	27.45 ± 1.23 aA	24.86 ± 0.50 aAB	23.03 ± 0.82 bA	15.13 ± 0.97 C	$p < 0.01$
		TSKC1-144	22.98 ± 0.86 B	23.98 ± 0.88 AB	24.58 ± 1.14 abAB	27.06 ± 0.96 aA	26.07 ± 1.33 aAB	14.45 ± 1.36 C	$p < 0.01$
		KDML105	22.82 ± 1.11 B	24.98 ± 0.62 AB	26.65 ± 1.59 aA	26.18 ± 1.50 aA	25.72 ± 1.12 aA	13.59 ± 0.54 C	$p < 0.01$
		[1] Critical-*p* value	ns	ns	$p < 0.05$	$p < 0.01$	$p < 0.01$	ns	
		**mean**	**23.29 ± 0.38 bC**	**24.57 ± 0.45 ABC**	**25.79 ± 0.71 aA**	**24.81 ± 0.74 aAB**	**23.75 ± 0.77 aBC**	**14.08 ± 0.41 bD**	**$p < 0.01$**
	Semi-saline	Pokkali	27.54 ± 0.52 abA (+19%)	24.56 ± 1.47 AB (+3%)	23.97 ± 0.67 B (+8%)	22.77 ± 0.86 BC (+17%)	20.82 ± 1.41 CD (+14%)	17.96 ± 0.53 aD (+35%)	$p < 0.01$
		RD73	28.89 ± 0.42 aA (+24%)	25.25 ± 1.16 B (-1%)	21.22 ± 0.38 C (-24%)	23.63 ± 0.97 BC (-11%)	24.98 ± 1.37 B (-3%)	17.53 ± 1.86 aD (+26%)	$p < 0.01$
		CSSL8-94	25.85 ± 0.61 bA (+7%)	25.82 ± 0.65 A (+6%)	21.41 ± 1.19 B (-22%)	22.66 ± 0.60 B (-9%)	23.69 ± 1.15 AB (+3%)	15.90 ± 0.85 abC (+5%)	$p < 0.01$
		TSKC1-144	28.92 ± 0.86 aA (+26%)	25.95 ± 0.95 AB (+8%)	22.48 ± 1.12 CD (-9%)	21.69 ± 1.20 D (-20%)	25.61 ± 0.53 BC (-2%)	12.88 ± 1.23 bE (-11%)	$p < 0.01$
		KDML105	29.39 ± 0.32 aA (+29%)	25.41 ± 1.17 BC (+2%)	20.68 ± 0.46 D (-22%)	22.26 ± 0.64 CD (-15%)	25.65 ± 2.15 B (0%)	15.47 ± 0.21 abE (+14%)	$p < 0.01$
		[1] Critical-*p* value	$p < 0.01$	ns	ns	ns	ns	$p < 0.05$	
		**mean**	**28.12 ± 0.37 aA**	**25.40 ± 0.46 B**	**21.95 ± 0.43 bC**	**22.60 ± 0.38 bC**	**24.15 ± 0.70 aB**	**15.95 ± 0.60 aD**	**$p < 0.01$**
	Heavy-saline	Pokkali	25.93 ± 1.05 cAB (+12%)	22.83 ± 1.54 BC (-4%)	26.01 ± 1.28 A (+16%)	23.09 ± 0.99 ABC (+19%)	20.36 ± 1.15 C (+11%)	12.55 ± 0.96 D (-6%)	$p < 0.01$
		RD73	30.98 ± 0.68 abA (+33%)	28.86 ± 1.53 AB (+13%)	24.82 ± 0.82 BC (-11%)	23.37 ± 0.34 BC (-12%)	19.15 ± 3.46 C (-25%)	12.08 ± 2.42 D (-13%)	$p < 0.01$
		CSSL8-94	31.64 ± 1.20 aA (+31%)	26.63 ± 1.16 B (+9%)	24.37 ± 1.20 B (-11%)	24.90 ± 0.64 B (0%)	23.04 ± 2.08 B (0%)	11.06 ± 2.01 C (-27%)	$p < 0.01$
		TSKC1-144	26.43 ± 0.91 cA (+15%)	25.00 ± 1.04 A (+4%)	23.22 ± 0.80 A (-6%)	24.37 ± 0.47 A (-10%)	17.12 ± 1.57 B (-34%)	9.04 ± 1.54 C (-37%)	$p < 0.01$
		KDML105	28.28 ± 1.20 bcA (+29%)	26.21 ± 2.08 AB (+5%)	25.51 ± 1.44 AB (-4%)	26.74 ± 1.41 AB (+2%)	22.94 ± 0.74 B (-11%)	10.89 ± 1.06 C (-20%)	$p < 0.01$
		[1] Critical-*p* value	$p < 0.01$	ns	ns	ns	ns	ns	
		**mean**	**28.65 ± 0.67 aA**	**25.91 ± 0.75 B**	**24.79 ± 0.50 aB**	**24.49 ± 0.45 aB**	**20.52 ± 0.96 bC**	**11.12 ± 0.73 cD**	**$p < 0.01$**
		**[3] Critical-*p* value**	**$p < 0.01$**	**ns**	**$p < 0.01$**	**$p < 0.05$**	**$p < 0.01$**	**$p < 0.01$**	
gs	Non-saline	Pokkali	0.15 ± 0.01 aC	0.57 ± 0.05 A	0.45 ± 0.04 cB	0.39 ± 0.03 B	0.41 ± 0.04 abB	0.14 ± 0.01 C	$p < 0.01$
		RD73	0.11 ± 0.01 abC	0.54 ± 0.02 AB	0.56 ± 0.04 abA	0.55 ± 0.03 AB	0.48 ± 0.03 aB	0.16 ± 0.02 C	$p < 0.01$
		CSSL8-94	0.09 ± 0.01 bC	0.52 ± 0.03 A	0.52 ± 0.03 abcA	0.40 ± 0.08 B	0.28 ± 0.04 cB	0.16 ± 0.01 C	$p < 0.01$
		TSKC1-144	0.12 ± 0.02 abC	0.48 ± 0.03 A	0.48 ± 0.02 bcA	0.41 ± 0.05 A	0.29 ± 0.03 cD	0.14 ± 0.03 C	$p < 0.01$
		KDML105	0.11 ± 0.02 abD	0.54 ± 0.03 AB	0.58 ± 0.02 aA	0.48 ± 0.07 B	0.37 ± 0.03 bcC	0.14 ± 0.02 D	$p < 0.01$
		[1] Critical-*p* value	$p < 0.05$	ns	$p < 0.05$	ns	$p < 0.01$	ns	
		**mean**	**0.12 ± 0.01 bD**	**0.53 ± 0.01 aA**	**0.52 ± 0.02 aA**	**0.44 ± 0.03 B**	**0.37 ± 0.02 aC**	**0.15 ± 0.01 aD**	**$p < 0.01$**
	Semi-saline	Pokkali	0.49 ± 0.01 A	0.16 ± 0.01 C	0.14 ± 0.02 bC	0.34 ± 0.11 AB	0.49 ± 0.03 A	0.18 ± 0.03 BC	$p < 0.01$
		RD73	0.50 ± 0.03 AB	0.16 ± 0.02 C	0.51 ± 0.03 aAB	0.56 ± 0.01 A	0.44 ± 0.02 B	0.19 ± 0.04 C	$p < 0.01$
		CSSL8-94	0.41 ± 0.02 BC	0.17 ± 0.01 D	0.52 ± 0.01 aA	0.49 ± 0.01 AB	0.38 ± 0.06 C	0.17 ± 0.01 D	$p < 0.01$

Parameter	Saline field	Rice genotype	Early vegetative	Stem elongation	Early booting	Flowering	Milky	Mature	[2] Critical-p value
		TSKC1-144	0.48 ± 0.05 A	0.14 ± 0.01 C	0.52 ± 0.06 aA	0.49 ± 0.03 A	0.31 ± 0.03 B	0.10 ± 0.01 C	$p < 0.01$
		KDML105	0.50 ± 0.04 A	0.16 ± 0.01 C	0.49 ± 0.01 aAB	0.46 ± 0.05 AB	0.38 ± 0.07 B	0.12 ± 0.01 C	$p < 0.01$
		[1] Critical-p value	ns	ns	$p < 0.01$	ns	ns	ns	
		**mean**	**0.48 ± 0.02 aA**	**0.16 ± 0.01 bC**	**0.44 ± 0.04 bAB**	**0.47 ± 0.03 A**	**0.40 ± 0.02 aB**	**0.15 ± 0.01 aC**	**$p < 0.01$**
	**Heavy-saline**	Pokkali	0.39 ± 0.09 A	0.10 ± 0.01 cB	0.11 ± 0.01 cB	0.27 ± 0.10 bAB	0.42 ± 0.04 abA	0.12 ± 0.02 B	$p < 0.01$
		RD73	0.53 ± 0.07 A	0.13 ± 0.01 aBC	0.47 ± 0.02 abA	0.45 ± 0.02 aA	0.23 ± 0.06 abB	0.10 ± 0.02 C	$p < 0.01$
		CSSL8-94	0.62 ± 0.01 A	0.12 ± 0.01 abD	0.50 ± 0.04 aB	0.49 ± 0.02 aB	0.30 ± 0.06 aC	0.11 ± 0.02 D	$p < 0.01$
		TSKC1-144	0.38 ± 0.04 B	0.11 ± 0.01 bcCD	0.41 ± 0.01 bAB	0.43 ± 0.02 aA	0.15 ± 0.01 bB	0.08 ± 0.02 C	$p < 0.01$
		KDML105	0.45 ± 0.07 A	0.12 ± 0.01 abC	0.48 ± 0.03 aA	0.49 ± 0.02 aA	0.27 ± 0.02 aB	0.10 ± 0.02 C	$p < 0.01$
		[1] Critical-p value	ns	$p < 0.05$	$p < 0.01$	$p < 0.05$	$p < 0.01$	ns	
		**mean**	**0.47 ± 0.03 aA**	**0.12 ± 0.01 cD**	**0.39 ± 0.03 bB**	**0.43 ± 0.03 AB**	**0.28 ± 0.03 bC**	**0.10 ± 0.01 bD**	**$p < 0.01$**
		**[3] Critical-p value**	**$p < 0.01$**	**$p < 0.01$**	**$p < 0.05$**	ns	**$p < 0.01$**	**$p < 0.01$**	
**T_r**	**Non-saline**	Pokkali	6.69 ± 0.50 aBC	10.41 ± 0.27 A	7.13 ± 0.37 B	5.72 ± 0.55 CD	5.06 ± 0.27 cD	3.35 ± 0.23 aE	$p < 0.01$
		RD73	4.70 ± 0.20 bC	10.28 ± 0.42 A	8.01 ± 0.51 B	7.65 ± 0.56 B	7.37 ± 0.35 aB	7.00 ± 0.52 bB	$p < 0.01$
		CSSL8-94	4.31 ± 0.37 bD	10.36 ± 0.56 A	7.51 ± 0.39 B	6.72 ± 0.70 BC	5.65 ± 0.55 bcCD	7.08 ± 0.51 bBC	$p < 0.01$
		TSKC1-144	5.07 ± 0.79 abBC	9.12 ± 0.50 A	7.21 ± 0.14 B	6.68 ± 0.51 BC	5.58 ± 0.52 bcC	6.34 ± 0.94 bBC	$p < 0.01$
		KDML105	4.64 ± 0.70 bD	10.63 ± 0.30 A	8.67 ± 0.08 B	7.77 ± 0.63 BC	6.64 ± 0.23 abC	6.46 ± 0.61 bC	$p < 0.01$
		[1] Critical-p value	$p < 0.01$	ns	$p < 0.05$	ns	$p < 0.01$	$p < 0.01$	
		**mean**	**5.24 ± 0.27 bE**	**10.16 ± 0.21 aA**	**7.71 ± 0.19 aB**	**6.91 ± 0.29 aC**	**6.06 ± 0.25 D**	**6.05 ± 0.40 aD**	**$p < 0.05$**
	**Semi-saline**	Pokkali	8.79 ± 0.31 A	4.48 ± 0.26 C	4.02 ± 0.31 bC	5.50 ± 0.85 BC	6.40 ± 0.33 B	4.90 ± 0.88 cBC	$p < 0.01$
		RD73	9.19 ± 0.22 A	4.64 ± 0.52 C	6.63 ± 0.10 aB	6.95 ± 0.15 B	7.02 ± 0.24 B	7.83 ± 1.16 aC	$p < 0.01$
		CSSL8-94	8.09 ± 0.21 A	4.58 ± 0.19 C	6.63 ± 0.16 aB	6.56 ± 0.07 B	6.30 ± 0.67 B	7.18 ± 0.43 abAB	$p < 0.01$
		TSKC1-144	9.07 ± 0.54 A	4.01 ± 0.20 D	6.66 ± 0.27 aB	6.58 ± 0.20 B	5.81 ± 0.31 BC	4.75 ± 0.52 cCD	$p < 0.01$
		KDML105	9.27 ± 0.19 A	4.82 ± 0.46 C	6.62 ± 0.13 aB	6.25 ± 0.31 B	6.30 ± 0.66 B	5.76 ± 0.25 bcBC	$p < 0.01$
		[1] Critical-p value	ns	ns	$p < 0.01$	ns	ns	$p < 0.05$	
		**mean**	**8.88 ± 0.16 aA**	**4.51 ± 0.15 bC**	**6.11 ± 0.25 bB**	**6.37 ± 0.20 aB**	**6.37 ± 0.21 B**	**6.08 ± 0.40 aB**	**$p < 0.01$**
	**Heavy-saline**	Pokkali	7.23 ± 0.97 A	3.04 ± 0.11 cC	3.16 ± 0.11 bC	4.41 ± 0.85 bBC	5.58 ± 0.35 AB	3.31 ± 0.33 C	$p < 0.01$
		RD73	9.01 ± 0.51 A	3.58 ± 0.10 aC	5.97 ± 0.15 aB	5.88 ± 0.15 aB	5.24 ± 1.15 BC	4.86 ± 0.92 BC	$p < 0.01$
		CSSL8-94	9.81 ± 0.13 A	3.42 ± 0.15 abD	6.23 ± 0.22 aB	6.16 ± 0.22 aB	6.45 ± 0.41 B	4.91 ± 0.86 C	$p < 0.01$
		TSKC1-144	7.51 ± 0.48 A	3.13 ± 0.06 bcC	5.67 ± 0.09 aB	5.81 ± 0.07 aB	3.83 ± 0.27 C	4.02 ± 0.78 C	$p < 0.01$
		KDML105	8.45 ± 0.73 A	3.43 ± 0.14 abC	6.16 ± 0.34 aB	6.27 ± 0.22 aB	6.18 ± 0.56 B	4.88 ± 0.91 BC	$p < 0.01$
		[1] Critical-p value	ns	$p < 0.05$	$p < 0.01$	$p < 0.05$	ns	ns	
		**mean**	**8.40 ± 0.33 aA**	**3.32 ± 0.07 cD**	**5.44 ± 0.28 cB**	**5.71 ± 0.22 bB**	**5.45 ± 0.33 B**	**4.40 ± 0.35 bC**	**$p < 0.01$**
		**[3] Critical-p value**	**$p < 0.01$**	**$p < 0.01$**	**$p < 0.01$**	**$p < 0.01$**	**ns**	**$p < 0.01$**	
**WUE**	**Non-saline**	Pokkali	3.53 ± 0.35 bAB	2.29 ± 0.04 cC	3.11 ± 0.06 B	3.46 ± 0.27 AB	3.64 ± 0.19 bAB	4.03 ± 0.35 aA	$p < 0.01$
		RD73	4.95 ± 0.18 abA	2.49 ± 0.08 abC	3.54 ± 0.31 B	3.53 ± 0.32 B	3.49 ± 0.16 bB	1.99 ± 0.14 bC	$p < 0.01$
		CSSL8-94	5.72 ± 0.46 abA	2.35 ± 0.04 bcC	3.67 ± 0.20 B	3.80 ± 0.35 B	4.14 ± 0.28 abB	2.14 ± 0.09 bC	$p < 0.01$
		TSKC1-144	3.96 ± 0.29 abAB	2.63 ± 0.05 aCD	3.40 ± 0.09 BC	4.13 ± 0.40 AB	4.73 ± 0.29 aA	2.33 ± 0.14 bD	$p < 0.01$
		KDML105	5.30 ± 0.93 abA	2.34 ± 0.01 bcC	3.07 ± 0.20 BC	3.46 ± 0.44 B	3.88 ± 0.23 bB	2.14 ± 0.17 bC	$p < 0.01$
		[1] Critical-p value	$p < 0.05$	$p < 0.01$	ns	ns	$p < 0.05$	$p < 0.01$	
		**mean**	**4.69 ± 0.28 aA**	**2.42 ± 0.03 cD**	**3.36 ± 0.09 bC**	**3.68 ± 0.16 bBC**	**3.98 ± 0.14 B**	**2.53 ± 0.19 D**	**$p < 0.05$**
	**Semi-saline**	Pokkali	3.14 ± 0.13 C	5.52 ± 0.40 AB	6.06 ± 0.48 aA	4.49 ± 0.81 BC	3.24 ± 0.12 cC	3.94 ± 0.55 aC	$p < 0.01$

Parameter	Saline field	Rice genotype	Early vegetative	Stem elongation	Early booting	Flowering	Milky	Mature	[2] Critical-p value
		RD73	3.14 ± 0.03 B	5.57 ± 0.41 A	3.20 ± 0.09 bB	3.39 ± 0.07 B	3.57 ± 0.26 bcB	2.29 ± 0.19 bC	$p < 0.01$
		CSSL8-94	3.20 ± 0.16 C	5.66 ± 0.27 A	3.22 ± 0.15 bBC	3.45 ± 0.10 BC	3.85 ± 0.33 bB	2.22 ± 0.07 bD	$p < 0.01$
		TSKC1-144	3.21 ± 0.19 C	6.54 ± 0.57 A	3.37 ± 0.06 bC	3.28 ± 0.13 C	4.44 ± 0.26 aB	2.74 ± 0.15 bC	$p < 0.01$
		KDML105	3.17 ± 0.03 CD	5.36 ± 0.40 A	3.12 ± 0.07 bCD	3.59 ± 0.25 BC	4.09 ± 0.10 abB	2.70 ± 0.15 bD	$p < 0.01$
		[1] Critical-p value	ns	ns	$p < 0.01$	ns	$p < 0.05$	$p < 0.01$	
		**mean**	**3.17 ± 0.05 bCD**	**5.73 ± 0.19 bA**	**3.79 ± 0.28 bB**	**3.64 ± 0.18 bBC**	**3.84 ± 0.13 B**	**2.78 ± 0.18 D**	**$p < 0.01$**
	**Heavy-saline**	Pokkali	3.73 ± 0.37 C	7.48 ± 0.37 AB	8.21 ± 0.12 aA	5.92 ± 1.23 B	3.65 ± 0.13 C	3.82 ± 0.18 aC	$p < 0.01$
		RD73	3.46 ± 0.18 C	8.04 ± 0.33 A	4.15 ± 0.12 bB	3.97 ± 0.06 BC	3.83 ± 0.30	2.55 ± 0.37 bD	$p < 0.01$
		CSSL8-94	3.21 ± 0.08 C	7.81 ± 0.42 A	3.90 ± 0.09 bB	4.05 ± 0.20 B	3.55 ± 0.14 BC	2.25 ± 0.10 bD	$p < 0.01$
		TSKC1-144	3.54 ± 0.17 C	7.97 ± 0.34 A	4.09 ± 0.09 bBC	4.19 ± 0.05 B	4.44 ± 0.19 B	2.27 ± 0.16 bD	$p < 0.01$
		KDML105	3.41 ± 0.28 C	7.60 ± 0.31 A	4.14 ± 0.11 bBC	4.26 ± 0.17 B	3.77 ± 0.25 BC	2.38 ± 0.32 bD	$p < 0.01$
		[1] Critical-p value	ns	ns	$p < 0.01$	ns	ns	$p < 0.05$	
		**mean**	**3.47 ± 0.10 bC**	**7.78 ± 0.15 aA**	**4.90 ± 0.38 aB**	**4.48 ± 0.28 aB**	**3.85 ± 0.11 C**	**2.65 ± 0.17 D**	**$p < 0.01$**
		**[3] Critical-p value**	**$p < 0.01$**	**$p < 0.01$**	**$p < 0.01$**	**$p < 0.05$**	**ns**	**ns**	
**ΦPSII**	**Non-saline**	Pokkali	0.32 ± 0.01 A	0.24 ± 0.01 B	0.24 ± 0.01 B	0.25 ± 0.01 B	0.25 ± 0.01 ab B	0.15 ± 0.01 C	$p < 0.01$
		RD73	0.32 ± 0.01 A	0.26 ± 0.01 BC	0.31 ± 0.02 AB	0.25 ± 0.03 C	0.23 ± 0.01 bC	0.16 ± 0.01 D	$p < 0.01$
		CSSL8-94	0.30 ± 0.01 A	0.27 ± 0.02 AB	0.31 ± 0.02 A	0.23 ± 0.03 B	0.17 ± 0.01 cC	0.17 ± 0.01 C	$p < 0.01$
		TSKC1-144	0.32 ± 0.01 A	0.27 ± 0.01 BC	0.30 ± 0.01 AB	0.29 ± 0.01 AB	0.25 ± 0.01 abC	0.16 ± 0.02 D	$p < 0.01$
		KDML105	0.30 ± 0.01 A	0.27 ± 0.01 A	0.27 ± 0.02 A	0.28 ± 0.02 A	0.27 ± 0.01 aA	0.19 ± 0.01 B	$p < 0.01$
		[1] Critical-p value	ns	ns	ns	ns	$p < 0.01$	ns	
		**mean**	**0.31 ± 0.01 aA**	**0.26 ± 0.01 bC**	**0.29 ± 0.01 aB**	**0.26 ± 0.01 abC**	**0.23 ± 0.01 D**	**0.16 ± 0.01 aE**	**$p < 0.01$**
	**Semi-saline**	Pokkali	0.29 ± 0.01 A	0.25 ± 0.02 A	0.28 ± 0.03 A	0.24 ± 0.02 A	0.24 ± 0.02 A	0.16 ± 0.01 B	$p < 0.01$
		RD73	0.31 ± 0.01 A	0.26 ± 0.02 B	0.22 ± 0.01 B	0.25 ± 0.02 B	0.24 ± 0.02 B	0.16 ± 0.01 C	$p < 0.01$
		CSSL8-94	0.28 ± 0.01 A	0.27 ± 0.01 AB	0.24 ± 0.01 BC	0.24 ± 0.01 BC	0.23 ± 0.01 C	0.15 ± 0.01 D	$p < 0.01$
		TSKC1-144	0.31 ± 0.01 A	0.28 ± 0.02 AB	0.24 ± 0.01 B	0.23 ± 0.01 B	0.27 ± 0.02 AB	0.12 ± 0.01 C	$p < 0.01$
		KDML105	0.30 ± 0.01 A	0.28 ± 0.02 AB	0.22 ± 0.01 C	0.24 ± 0.01 BC	0.27 ± 0.02 AB	0.17 ± 0.02 D	$p < 0.01$
		[1] Critical-p value	ns	ns	ns	ns	ns	ns	
		**mean**	**0.30 ± 0.01 abA**	**0.27 ± 0.01 bB**	**0.24 ± 0.01 bC**	**0.24 ± 0.01 bC**	**0.25 ± 0.01 BC**	**0.15 ± 0.01 abD**	**$p < 0.01$**
	**Heavy-saline**	Pokkali	0.26 ± 0.01 bCD	0.30 ± 0.01 AB	0.34 ± 0.01 aA	0.28 ± 0.03 BC	0.23 ± 0.01 D	0.14 ± 0.01 E	$p < 0.01$
		RD73	0.30 ± 0.01 aAB	0.34 ± 0.02 A	0.29 ± 0.01 bB	0.27 ± 0.01 B	0.22 ± 0.03 C	0.15 ± 0.02 D	$p < 0.01$
		CSSL8-94	0.31 ± 0.01 aAB	0.32 ± 0.01 A	0.26 ± 0.01 bBC	0.27 ± 0.01 BC	0.25 ± 0.03 C	0.15 ± 0.01 D	$p < 0.01$
		TSKC1-144	0.28 ± 0.01 abB	0.34 ± 0.02 A	0.27 ± 0.01 bB	0.28 ± 0.01 B	0.21 ± 0.02 C	0.11 ± 0.01 D	$p < 0.01$
		KDML105	0.30 ± 0.01 aA	0.33 ± 0.02 A	0.29 ± 0.01 bAB	0.29 ± 0.02 AB	0.24 ± 0.01 B	0.15 ± 0.03 C	$p < 0.01$
		[1] Critical-p value	$p < 0.05$	ns	$p < 0.01$	ns	ns	ns	
		**mean**	**0.29 ± 0.01 bB**	**0.33 ± 0.01 aA**	**0.29 ± 0.01 aB**	**0.28 ± 0.01 aB**	**0.23 ± 0.01 C**	**0.14 ± 0.01 bD**	**$p < 0.01$**
		**[3] Critical-p value**	**$p < 0.05$**	**$p < 0.01$**	**$p < 0.01$**	**$p < 0.01$**	**ns**	**$p < 0.05$**	
**Fv'/Fm'**	**Non-saline**	Pokkali	0.48 ± 0.01 b B	0.53 ± 0.02 a-cA	0.47 ± 0.02 cB	0.46 ± 0.02 cB	0.50 ± 0.01 bcAB	0.34 ± 0.02 C	$p < 0.01$
		RD73	0.52 ± 0.02 abA	0.58 ± 0.01 aA	0.57 ± 0.01 abA	0.58 ± 0.01 aA	0.56 ± 0.02 aA	0.40 ± 0.04 B	$p < 0.01$
		CSSL8-94	0.53 ± 0.01 aA	0.54 ± 0.03 abA	0.57 ± 0.01 abA	0.57 ± 0.01 abA	0.55 ± 0.01 abA	0.40 ± 0.03 B	$p < 0.01$
		TSKC1-144	0.48 ± 0.01 bA	0.49 ± 0.01 cA	0.53 ± 0.02 bA	0.51 ± 0.03 bcA	0.49 ± 0.02 cA	0.33 ± 0.03 B	$p < 0.01$
		KDML105	0.49 ± 0.02 abB	0.52 ± 0.01 bcB	0.61 ± 0.02 aA	0.56 ± 0.03 abAB	0.50 ± 0.03 bcB	0.39 ± 0.02 C	$p < 0.01$
		[1] Critical-p value	$p < 0.05$	$p < 0.05$	$p < 0.01$	$p < 0.01$	$p < 0.05$	ns	
		**mean**	**0.50 ± 0.01 cC**	**0.53 ± 0.01 bAB**	**0.55 ± 0.01 cA**	**0.53 ± 0.01 bAB**	**0.52 ± 0.01 BC**	**0.37 ± 0.01 cD**	**$p < 0.01$**

Parameter	Saline field	Rice genotype	Early vegetative	Stem elongation	Early booting	Flowering	Milky	Mature	[2] Critical-p value
	Semi-saline	Pokkali	0.58 ± 0.01 A	0.56 ± 0.01 bcA	0.56 ± 0.02 cA	0.57 ± 0.02 bA	0.58 ± 0.03 A	0.44 ± 0.01 B	$p < 0.01$
		RD73	0.57 ± 0.02 BC	0.60 ± 0.02 abABC	0.62 ± 0.02 aAB	0.63 ± 0.01 aA	0.56 ± 0.03 CD	0.52 ± 0.03 D	$p < 0.05$
		CSSL8-94	0.57 ± 0.01 AB	0.61 ± 0.02 aA	0.58 ± 0.01 bcAB	0.62 ± 0.01 aA	0.54 ± 0.03 BC	0.50 ± 0.03 C	$p < 0.01$
		TSKC1-144	0.58 ± 0.02 A	0.52 ± 0.01 cB	0.62 ± 0.01 abA	0.61 ± 0.01 aA	0.51 ± 0.01 B	0.40 ± 0.03 C	$p < 0.01$
		KDML105	0.59 ± 0.01 A	0.60 ± 0.01 abA	0.63 ± 0.01 aA	0.62 ± 0.01 aA	0.53 ± 0.02 B	0.49 ± 0.03 B	$p < 0.01$
		[1] Critical-p value	ns	$p < 0.01$	$p < 0.01$	$p < 0.05$	ns	ns	
		**mean**	**0.58 ± 0.01 aB**	**0.58 ± 0.01 aB**	**0.60 ± 0.01 aAB**	**0.61 ± 0.01 aA**	**0.54 ± 0.01 C**	**0.47 ± 0.02 aD**	**$p < 0.01$**
	Heavy-saline	Pokkali	0.52 ± 0.01 bcBC	0.49 ± 0.01 cC	0.52 ± 0.01 cBC	0.54 ± 0.02 bAB	0.57 ± 0.01 aA	0.38 ± 0.02 bD	$p < 0.01$
		RD73	0.55 ± 0.01 abB	0.57 ± 0.01 aB	0.61 ± 0.01 aA	0.62 ± 0.01 aA	0.50 ± 0.02 bcC	0.50 ± 0.02 aC	$p < 0.01$
		CSSL8-94	0.57 ± 0.01 aB	0.54 ± 0.01 abBC	0.61 ± 0.01 aA	0.61 ± 0.01 aA	0.52 ± 0.02 abC	0.40 ± 0.01 bD	$p < 0.01$
		TSKC1-144	0.51 ± 0.02 cBC	0.51 ± 0.01 bcBC	0.56 ± 0.01 bAB	0.58 ± 0.01 aA	0.46 ± 0.02 cC	0.40 ± 0.04 bD	$p < 0.01$
		KDML105	0.56 ± 0.02 aBC	0.54 ± 0.01 abBC	0.59 ± 0.01 aAB	0.62 ± 0.01 aA	0.53 ± 0.01 abC	0.45 ± 0.03 abD	$p < 0.01$
		[1] Critical-p value	$p < 0.05$	$p < 0.01$	$p < 0.01$	$p < 0.01$	$p < 0.05$	$p < 0.05$	
		**mean**	**0.54 ± 0.01 bB**	**0.53 ± 0.01 bBC**	**0.58 ± 0.01 bA**	**0.59 ± 0.01 aA**	**0.52 ± 0.01 C**	**0.43 ± 0.01 bD**	**$p < 0.01$**
		**[3] Critical-p value**	**$p < 0.01$**	**$p < 0.01$**	**$p < 0.01$**	**$p < 0.01$**	**ns**	**$p < 0.01$**	
ETR (μmol e⁻ m⁻² s⁻¹)	Non-saline	Pokkali	172 ± 1 A	129 ± 8 B	129 ± 6 B	132 ± 7 B	135 ± 7 abB	80 ± 5 C	$p < 0.01$
		RD73	172 ± 6 A	138 ± 5 BC	164 ± 12 AB	133 ± 15 C	123 ± 6 bC	85 ± 6 D	$p < 0.01$
		CSSL8-94	161 ± 8 A	146 ± 9 AB	162 ± 9 A	122 ± 15 B	90 ± 5 cC	91 ± 5 C	$p < 0.01$
		TSKC1-144	169 ± 4 B	146 ± 4 AB	158 ± 4 AB	154 ± 6 A	132 ± 8 abAB	87 ± 8 C	$p < 0.01$
		KDML105	160 ± 7 A	146 ± 7 A	146 ± 11 A	147 ± 10 A	143 ± 6 aA	100 ± 2 B	$p < 0.01$
		[1] Critical-p value	ns	ns	ns	ns	$p < 0.01$	ns	
		**mean**	**167.28 ± 2.57 aA**	**141.39 ± 3.19 bC**	**152.47 ± 4.64 aB**	**138.18 ± 5.25 abC**	**125.04 ± 4.93 D**	**89.10 ± 2.78 aE**	**$p < 0.01$**
	Semi-saline	Pokkali	155 ± 7 A	132 ± 10 A	148 ± 15 A	130 ± 9 A	127 ± 12 A	87 ± 7 B	$p < 0.01$
		RD73	167 ± 7 A	136 ± 12 B	116 ± 5 B	133 ± 9 B	126 ± 12 B	87 ± 6 C	$p < 0.01$
		CSSL8-94	151 ± 5 A	144 ± 6 AB	128 ± 4 BC	129 ± 6 BC	121 ± 8 C	82 ± 8 D	$p < 0.01$
		TSKC1-144	164 ± 8 A	149 ± 10 AB	130 ± 6 B	125 ± 7 B	145 ± 9 AB	66 ± 7 C	$p < 0.01$
		KDML105	160 ± 2 A	149 ± 13 AB	117 ± 4 C	126 ± 7 BC	143 ± 9 AB	91 ± 9 D	$p < 0.01$
		[1] Critical-p value	ns	ns	ns	ns	ns	ns	
		**mean**	**159.76 ± 2.85 abA**	**142.52 ± 4.42 bB**	**128.38 ± 4.10 bC**	**129.08 ± 3.08 bC**	**132.90 ± 4.53 BC**	**83.04 ± 3.55 abD**	**$p < 0.01$**
	Heavy-saline	Pokkali	138 ± 5 bCD	161 ± 8 AB	180 ± 5 aA	150 ± 14 BC	124 ± 4 D	78 ± 2 E	$p < 0.01$
		RD73	162 ± 5 aAB	182 ± 8 A	155 ± 4 bB	146 ± 4 B	117 ± 15 C	81 ± 9 D	$p < 0.01$
		CSSL8-94	165 ± 6 aAB	173 ± 7 A	140 ± 7 bBC	144 ± 7 BC	135 ± 14 C	80 ± 7 D	$p < 0.01$
		TSKC1-144	151 ± 5 abB	183 ± 8 A	146 ± 7 bB	148 ± 5 B	111 ± 9 C	58 ± 5 D	$p < 0.01$
		KDML105	157 ± 4 aA	173 ± 9A	157 ± 6 bAB	153 ± 10AB	130 ± 6 B	80 ± 13 C	$p < 0.01$
		[1] Critical-p value	$p < 0.05$	ns	$p < 0.01$	ns	ns	ns	
		**mean**	**155.17 ± 2.94 bB**	**174.87 ± 3.68 aA**	**156.09 ± 3.89 aB**	**148.59 ± 3.49 aB**	**123.62 ± 4.54 C**	**75.85 ± 3.82 bD**	**$p < 0.01$**
		**[3] Critical-p value**	**$p < 0.05$**	**$p < 0.01$**	**$p < 0.01$**	**$p < 0.01$**	**ns**	**$p < 0.05$**	

[1] Critical-p value for testing each trait among genotypes within growth stage (the same column). [2] Critical-p value for testing each trait among growth stages of each genotype (the same row) and [3] critical-p value for testing each trait among salinity fields within growth stage**.** [Values in the brackets indicated percentage increase (+) or decrease (-) in the saline fields compared with that in the non-saline field]

Supplementary Table S3. Plant height and biomass of leaf, stem, root and total biomass at maturity of five rice genotypes including Pokkali, RD73, CSSL8-94, TSKC1-144 and KDML105 after grew under non-saline (control), semi-saline and heavy-saline field measured at four growth stage including early vegetative, stem elongation, flowering and mature stage. Means which are significantly different (p < 0.05) among genotypes for each saline field and field across genotypes are denoted by different lower-case letters. For each genotype means which are significantly different (p < 0.05) among growth stage are denoted with capital letters. Data shows mean of four replicates ± standard error (SE).

Parameter	Saline field	Rice genotype	Early vegetative	Stem elongation	Flowering	Mature	[2] Critical-p value
**Plant height** (cm)	**Non-saline**	Pokkali	40 ± 1 aD	137 ± 3 aC	155 ± 2 bB	178 ± 2 aA	$p > 0.01$
		RD73	34 ± 1 bD	106 ± 3 bcC	151 ± 3 bcB	163 ± 1 bA	$p > 0.01$
		CSSL8-94	40 ± 1 aC	102 ± 4 cB	152 ± 2 bcA	156 ± 1 bA	$p > 0.01$
		TSKC1-144	39 ± 1 aC	117 ± 3 bB	170 ± 1 aA	176 ± 2 aA	$p > 0.01$
		KDML105	39 ± 1 aC	102 ± 7 cB	147 ± 1 cA	156 ± 4 bA	$p > 0.01$
		[1] Critical-p value	$p > 0.05$	$p > 0.01$	$p > 0.01$	$p > 0.01$	
		**mean**	**38.925 D**	**113.15 aC**	**155.35 aB**	**166.2 aA**	**$p > 0.01$**
	**Semi-saline**	Pokkali	35 ± 1 cC	123 ± 6 aB	159 ± 7 aA	152 ± 1 abA	$p > 0.01$
		RD73	35 ± 1 cC	104 ± 1 bB	138 ± 3 bA	136 ± 8 cA	$p > 0.01$
		CSSL8-94	42 ± 1 aC	107 ± 2 bB	152 ± 3 abA	147 ± 5 bcA	$p > 0.01$
		TSKC1-144	39 ± 1 abC	102 ± 3 bB	167 ± 3 aA	167 ± 3 aA	$p > 0.01$
		KDML105	37 ± 1 bcC	104 ± 4 bB	140 ± 1 bA	132 ± 3 cA	$p > 0.01$
		[1] Critical-p value	$p > 0.05$	$p > 0.05$	$p > 0.01$	$p > 0.01$	
		**mean**	**38.05 C**	**108.55 aB**	**151.5 aA**	**146.95 bA**	**$p > 0.01$**
	**Heavy-saline**	Pokkali	33 ± 2 C	116 ± 3 aB	150 ± 3 aA	163 ± 6 aA	$p > 0.01$
		RD73	35 ± 1 C	80 ± 11 bcB	121 ± 10 bA	120 ± 17 cA	$p > 0.01$
		CSSL8-94	57 ± 15 C	77 ± 3 cB	125 ± 2 bA	121 ± 3 cA	$p > 0.01$
		TSKC1-144	38 ± 1 C	101 ± 11 abB	148 ± 10 aA	148 ± 9 abA	$p > 0.05$
		KDML105	38 ± 1 C	77 ± 6 cB	131 ± 3 bA	127 ± 1 bcA	$p > 0.01$
		[1] Critical-p value	ns	$p > 0.01$	$p > 0.01$	$p > 0.01$	
		**mean**	**40.69 C**	**90.50 bB**	**135.15 bA**	**136.15 bA**	**$p > 0.01$**
		**[3] Critical-p value**	**ns**	**$p > 0.01$**	**$p > 0.01$**	**$p > 0.01$**	
**Leaf biomass** (g_{DW} plant^{-1})	**Non-saline**	Pokkali	0.09 ± 0.01 aB	14.42 ± 1.93 bA	16.06 ± 1.58 bA	13.26 ± 0.69 cA	$p > 0.01$
		RD73	0.05 ± 0.01 cC	24.93 ± 2.51 aA	20.28 ± 1.19 bB	18.19 ± 0.70 bB	$p > 0.01$
		CSSL8-94	0.09 ± 0.01 abC	25.54 ± 1.63 aA	20.03 ± 2.33 bB	21.40 ± 1.35 abAB	$p > 0.01$
		TSKC1-144	0.07 ± 0.01 bcB	24.35 ± 2.04 aA	30.34 ± 5.19 aA	23.44 ± 1.01 aA	$p > 0.01$
		KDML105	0.06 ± 0.01 cC	24.40 ± 0.98 aB	30.37 ± 2.46 aA	21.79 ± 1.75 aB	$p > 0.01$
		[1] Critical-p value	$p > 0.01$	$p > 0.05$	$p > 0.01$	$p > 0.01$	
		**mean**	**0.07 C**	**22.73 aAB**	**23.41 aA**	**19.62 aB**	**$p > 0.01$**
	**Semi-saline**	Pokkali	0.06 ± 0.01 B	15.87 ± 2.10 A	21.89 ± 4.00 A	6.26 ± 0.76 cB	$p > 0.01$
		RD73	0.06 ± 0.01 D	9.22 ± 0.94 C	18.47 ± 1.13 A	12.16 ± 0.79 bcB	$p > 0.01$
		CSSL8-94	0.08 ± 0.01 C	15.13 ± 2.29 B	21.06 ± 1.49 A	23.89 ± 2.44 aA	$p > 0.01$
		TSKC1-144	0.08 ± 0.01 B	15.67 ± 2.76 AB	28.42 ± 9.81 A	14.76 ± 1.85 bAB	$p > 0.05$
		KDML105	0.07 ± 0.01 C	11.62 ± 1.29 B	25.73 ± 3.31 A	26.07 ± 2.91 aA	$p > 0.01$

Parameter	Saline field	Rice genotype	Early vegetative	Stem elongation	Flowering	Mature	[2] Critical-p value
		[1] Critical-p value	ns	ns	ns	$p > 0.01$	
		**mean**	**0.07 C**	**13.50 bB**	**23.11 aA**	**16.63 aB**	**$p > 0.01$**
	Heavy-saline	Pokkali	0.03 ± 0.01 B	16.44 ± 2.16 A	17.20 ± 1.87 A	13.43 ± 1.99 aA	$p > 0.01$
		RD73	0.06 ± 0.01 C	8.31 ± 2.41 B	14.60 ± 2.44 A	3.81 ± 0.54 b BC	$p > 0.01$
		CSSL8-94	0.08 ± 0.01 C	10.34 ± 1.58 B	18.54 ± 3.32 A	12.55 ± 1.33 aAB	$p > 0.01$
		TSKC1-144	0.07 ± 0.02 B	12.08 ± 1.68 A	19.71 ± 5.90 A	15.78 ± 3.58 aA	$p > 0.01$
		KDML105	0.07 ± 0.01 C	9.05 ± 0.80 B	16.28 ± 1.97 A	12.31 ± 0.36 aB	$p > 0.01$
		[1] Critical-p value	ns	ns	ns	$p > 0.01$	
		**mean**	**0.06 C**	**11.24 bB**	**17.26 bA**	**11.58 bB**	**$p > 0.01$**
		**[3] Critical-p value**	**ns**	**$p > 0.01$**	**$p > 0.05$**	**$p > 0.01$**	
Stem biomass (g_{DW} plant^{-1})	Non-saline	Pokkali	0.03 ± 0.01 C	12.34 ± 1.33 B	23.07 ± 3.53 cA	18.99 ± 1.20 cA	$p > 0.01$
		RD73	0.02 ± 0.01 C	18.53 ± 3.20 B	35.59 ± 3.04 bA	20.00 ± 1.12 cB	$p > 0.01$
		CSSL8-94	0.04 ± 0.01 C	19.09 ± 2.46 B	30.30 ± 3.16 bcA	28.33 ± 1.94 bA	$p > 0.01$
		TSKC1-144	0.03 ± 0.01 D	17.52 ± 3.29 C	54.01 ± 4.17 aA	33.57 ± 3.35 aB	$p > 0.01$
		KDML105	0.03 ± 0.01 D	19.05 ± 1.60 C	52.91 ± 2.63 aA	26.12 ± 2.15 bB	$p > 0.01$
		[1] Critical-p value	ns	ns	$p > 0.01$	$p > 0.01$	
		**mean**	**0.034 D**	**17.30 aC**	**39.17 aA**	**25.40 bB**	**$p > 0.01$**
	Semi-saline	Pokkali	0.01 ± 0.01 cB	18.45 ± 5.53 A	21.30 ± 8.25 bA	29.11 ± 0.93 bA	$p > 0.01$
		RD73	0.04 ± 0.01 bC	4.05 ± 0.46 C	22.56 ± 2.48 bB	30.38 ± 2.39 bA	$p > 0.01$
		CSSL8-94	0.05 ± 0.01 aC	13.95 ± 3.68 B	26.16 ± 1.80 bA	24.16 ± 1.52 bA	$p > 0.01$
		TSKC1-144	0.04 ± 0.01 bB	11.22 ± 1.70 B	46.62 ± 6.99 aA	48.20 ± 3.52 aA	$p > 0.01$
		KDML105	0.03 ± 0.01 bC	8.89 ± 1.36 B	22.36 ± 3.49 bA	26.07 ± 3.23 bA	$p > 0.01$
		[1] Critical-p value	$p > 0.01$	ns	$p > 0.05$	$p > 0.01$	
		**mean**	**0.03 C**	**11.31 bB**	**27.80 bA**	**31.59 aA**	**$p > 0.01$**
	Heavy-saline	Pokkali	0.02 ± 0.01 bB	21.17 ± 5.01 aA	17.99 ± 3.36 A	16.94 ± 3.08 A	$p > 0.01$
		RD73	0.03 ± 0.01 aC	5.85 ± 2.67 bBC	15.97 ± 5.04 AB	18.61 ± 5.49 A	$p > 0.05$
		CSSL8-94	0.03 ± 0.01 aD	5.80 ± 1.51 bC	21.44 ± 2.78 A	11.15 ± 0.57 B	$p > 0.01$
		TSKC1-144	0.03 ± 0.01 abC	10.70 ± 1.80 bBC	29.58 ± 6.76 A	16.42 ± 4.50 B	$p > 0.01$
		KDML105	0.02 ± 0.01 abD	4.36 ± 1.16 bC	15.90 ± 2.10 BA	11.10 ± 1.15 B	$p > 0.01$
		[1] Critical-p value	$p > 0.05$	$p > 0.05$	ns	ns	
		**mean**	**0.03 D**	**9.58 bC**	**20.18 bA**	**14.84 cB**	**$p > 0.01$**
		**[3] Critical-p value**	**ns**	**$p > 0.01$**	**$p > 0.01$**	**$p > 0.01$**	
Root biomass (g_{DW} plant^{-1})	Non-saline	Pokkali	0.03 ± 0.01 abC	26.44 ± 3.08 aA	10.71 ± 1.55 B	8.44 ± 1.54 B	$p > 0.01$
		RD73	0.02 ± 0.01 bC	9.24 ± 1.81 bB	15.46 ± 1.11 A	13.47 ± 0.22 A	$p > 0.01$
		CSSL8-94	0.05 ± 0.01 aC	14.67 ± 3.61 bAB	20.13 ± 1.75 A	8.24 ± 2.86 B	$p > 0.01$
		TSKC1-144	0.03 ± 0.01 bB	15.41 ± 3.61 bA	17.29 ± 5.51 A	4.54 ± 0.83 B	$p > 0.01$
		KDML105	0.04 ± 0.01 abC	31.46 ± 3.47 aA	14.07 ± 4.95 B	9.45 ± 2.96 BC	$p > 0.01$
		[1] Critical-p value	$p > 0.05$	$p > 0.01$	ns	ns	
		**mean**	**0.03 C**	**19.44 aA**	**15.53 aA**	**8.83 bB**	**$p > 0.01$**
	Semi-saline	Pokkali	0.03 ± 0.01 C	8.82 ± 1.06 B	16.02 ± 2.87 A	15.85 ± 2.13 A	$p > 0.01$
		RD73	0.06 ± 0.02 B	10.10 ± 5.12 A	10.13 ± 2.30 A	15.25 ± 2.71 A	$p > 0.05$
		CSSL8-94	0.03 ± 0.01 C	6.47 ± 0.51 B	9.36 ± 0.89 B	20.55 ± 2.56 A	$p > 0.01$
		TSKC1-144	0.06 ± 0.01 B	7.01 ± 1.71 B	24.79 ± 9.32 A	15.12 ± 3.02 AB	$p > 0.05$
		KDML105	0.03 ± 0.01 C	5.75 ± 1.43 B	20.22 ± 3.01 A	18.33 ± 1.56 A	$p > 0.01$

Parameter	Saline field	Rice genotype	Early vegetative	Stem elongation	Flowering	Mature	[2] Critical-*p* value
		[1] Critical-*p* value	ns	ns	ns	ns	
		**mean**	**0.04 C**	**7.63 bB**	**16.10 aA**	**17.02 aA**	**$p > 0.01$**
	Heavy-saline	Pokkali	0.01 ± 0.01 bB	9.56 ± 1.81 A	15.92 ± 4.22 A	10.86 ± 1.97 A	$p > 0.01$
		RD73	0.04 ± 0.01 aB	3.34 ± 1.53 AB	6.59 ± 2.89 AB	9.91 ± 3.70 A	$p > 0.05$
		CSSL8-94	0.03 ± 0.01 abC	4.18 ± 0.76 BC	7.87 ± 2.30 AB	11.90 ± 2.19 A	$p > 0.01$
		TSKC1-144	0.04 ± 0.01 aB	6.39 ± 1.49 AB	8.85 ± 3.32 A	14.18 ± 4.19 A	$p > 0.05$
		KDML105	0.03 ± 0.01 aC	4.89 ± 2.79 B	3.73 ± 0.59 BC	10.29 ± 0.98 A	$p > 0.01$
		[1] Critical-*p* value	$p > 0.05$	ns	ns	ns	
		**mean**	**0.03 C**	**5.67 bB**	**8.59 bAB**	**11.43 bA**	**$p > 0.01$**
		**[3] Critical-*p* value**	**ns**	**$p > 0.01$**	**$p > 0.01$**	**$p > 0.01$**	
**Total biomass** (g_{DW} plant^{-1})	**Non-saline**	Pokkali	0.17 ± 0.02 abC	53.20 ± 6.32 B	49.85 ± 5.01 cB	92.78 ± 7.82 bA	$p > 0.01$
		RD73	0.09 ± 0.01 cD	52.71 ± 4.72 C	71.33 ± 4.81 bB	139.67 ± 6.75 aA	$p > 0.01$
		CSSL8-94	0.19 ± 0.02 aC	59.30 ± 2.00 B	70.46 ± 4.80 bB	136.26 ± 6.34 aA	$p > 0.01$
		TSKC1-144	0.13 ± 0.02 bcD	57.29 ± 6.06 C	101.64 ± 9.26 aB	134.81 9.18 aA	$p > 0.01$
		KDML105	0.13 ± 0.02 bcD	74.91 ± 5.10 C	97.36 ± 4.82 aB	150.76 ± 9.06 aA	$p > 0.01$
		[1] Critical-*p* value	$p > 0.05$	ns	$p > 0.01$	$p > 0.01$	
		**mean**	**0.14 D**	**59.48 aC**	**78.13 aB**	**130.86 bA**	**$p > 0.01$**
	**Semi-saline**	Pokkali	0.12 ± 0.01 C (-29%)	43.14 ± 8.48 B (-19%)	59.22 ± 9.67 B (+19%)	139.98 ± 6.07 A (+51%)	$p > 0.01$
		RD73	0.16 ± 0.03 D (+78%)	23.37 ± 5.89 C (-56%)	51.16 ± 4.87 B (-28%)	173.94 ± 10.20 A (+24%)	$p > 0.01$
		CSSL8-94	0.17 ± 0.01 D (-11%)	35.56 ± 6.07 C (-40%)	56.60 ± 0.31 B (-20%)	157.53 ± 7.25 A (+15%)	$p > 0.01$
		TSKC1-144	0.18 ± 0.02 C (+38%)	33.91 ± 5.75 C (-41%)	99.83 ± 24.98 B (-2%)	159.53 ± 10.60 A (+18%)	$p > 0.01$
		KDML105	0.13 ± 0.01 C (0%)	26.27 ± 3.78 C (-65%)	68.32 ± 8.80 B (-30%)	167.89 ± 17.08 A (+11%)	$p > 0.01$
		[1] Critical-*p* value	ns	ns	ns	ns	
		**mean**	**0.15 D**	**32.45 bC**	**67.02 aB**	**159.78 aA**	**$p > 0.01$**
	**Heavy-saline**	Pokkali	0.07 ± 0.01 bC (-59%)	47.17 ± 8.51 aB (-11%)	51.12 ± 9.27 B (+3%)	117.08 ± 15.94 A (+26%)	$p > 0.01$
		RD73	0.14 ± 0.01 aB (+56%)	17.51 ± 6.54 bB (-67%)	37.17 ± 9.56 AB (-48%)	71.19 ± 26.41 A (-48%)	$p > 0.05$
		CSSL8-94	0.14 ± 0.01 aD (-26%)	20.33 ± 3.63 bC (-66%)	47.86 ± 7.83 B (-32%)	84.29 ± 6.72 A (-38%)	$p > 0.01$
		TSKC1-144	0.15 ± 0.03 aC (+15%)	29.18 ± 3.7 bBC (-49%)	58.16 ± 14.29 AB (-43%)	85.03 ± 21.95 A (-36%)	$p > 0.01$
		KDML105	0.13 ± 0.01 aC (0%)	18.31 ± 4.3 bBC(-76%)	35.92 ± 3.92 B (-63%)	82.10 ± 12.35 A (-45%)	$p > 0.01$
		[1] Critical-*p* value	$p > 0.05$	$p > 0.05$	ns	ns	
		**mean**	**0.12 D**	**26.50 bC**	**46.04 bB**	**87.94 cA**	**$p > 0.01$**
		**[3] Critical-*p* value**	**ns**	**$p > 0.01$**	**$p > 0.01$**	**$p > 0.01$**	

[1] Critical-*p* value for testing each trait among genotypes within growth stage (the same column). [2] Critical-*p* value for testing each trait among growth stages of each genotype (the same row) and [3] critical-*p* value for testing each trait among salinity fields within growth stage. [Values in the brackets indicated percentage increase (+) or decrease (-) in the saline fields compared with that in the non-saline field]

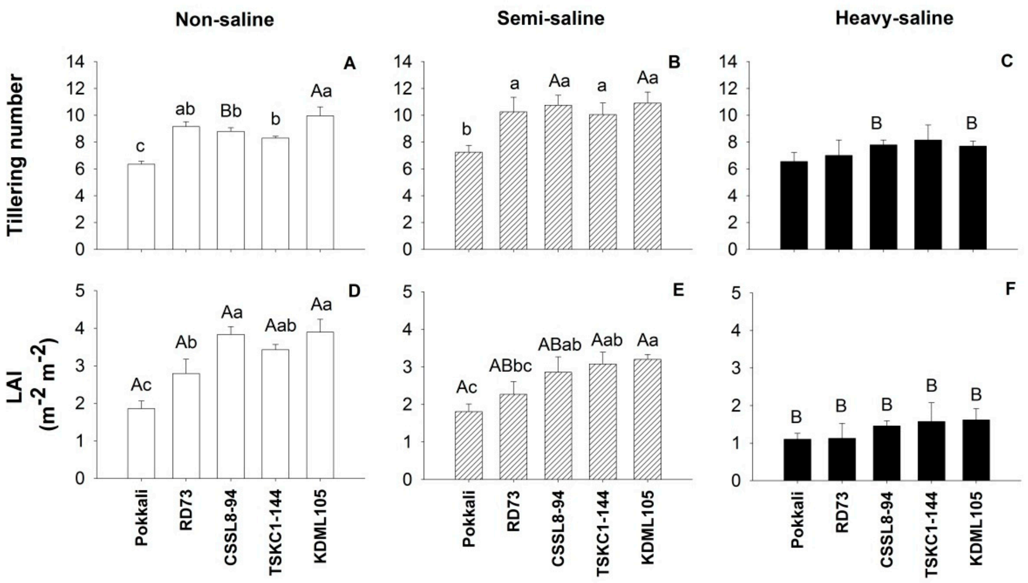

Figure S1. Tiller number and leaf area index (LAI) of five rice genotypes including Pokkali, RD73, CSSL8-94, TSKC1-144 and KDML105 after grew under non-saline (**A**,**D**), semi-saline (**B**,**E**) and heavy-saline fields (**C**,**F**) measured at mature stage. Means which are significantly different (p < 0.05) among genotypes for each saline field are denoted by different lower-case letters. For each genotype means which are significantly different (p < 0.05) among saline fields are denoted with capital letters. Data shows mean of four replicates ± standard error (SE) (n=4).

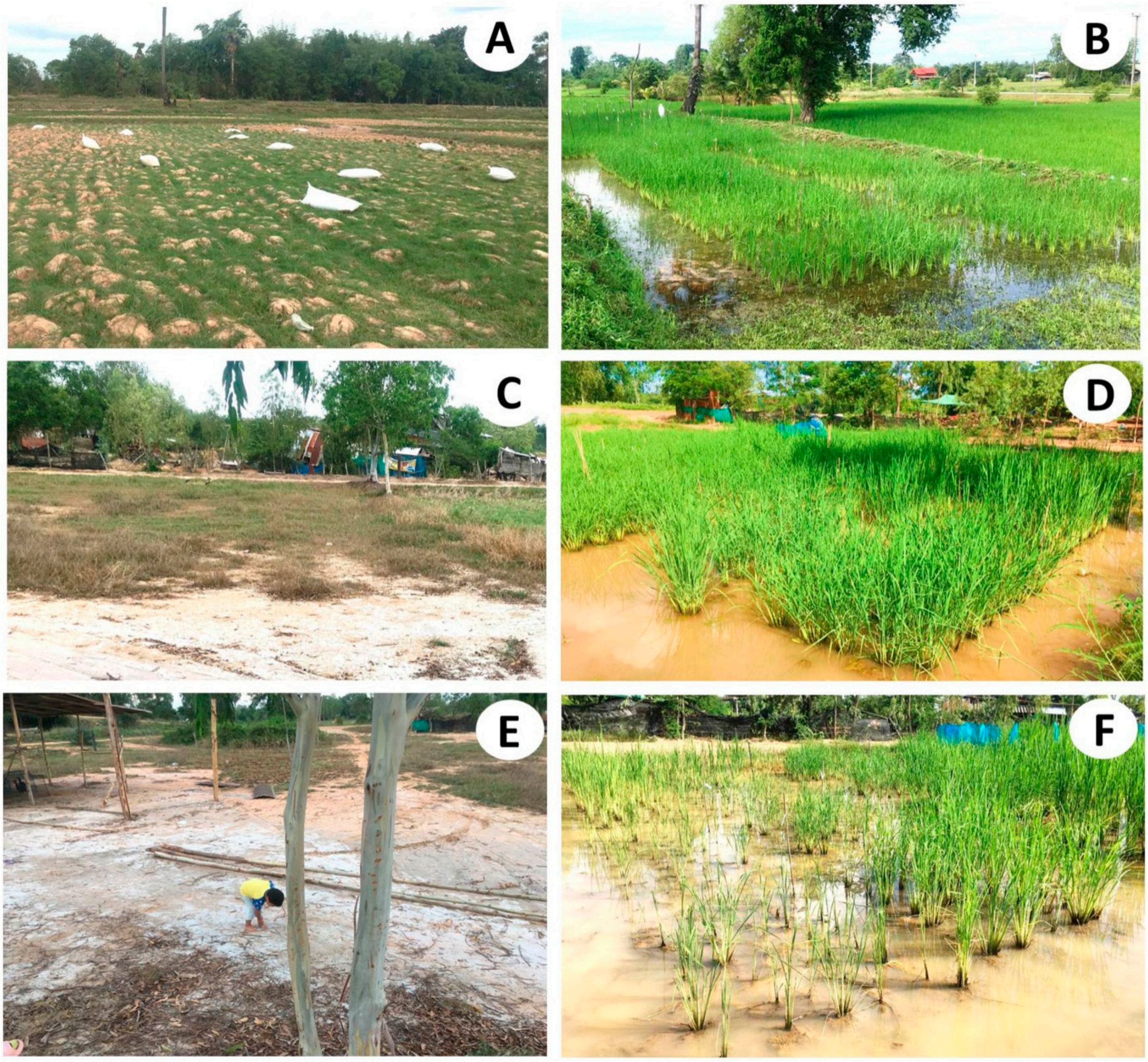

Figure S2. Experimental fields before land preparation; non-saline (**A**)**,** semi-saline **(C)** and heavy-saline **(E),** and rice growing in the non-saline (**B**), semi-saline (**D**) and heavy-saline field (**F**). For the semi-saline and heavy-saline plots, famers avoided growing rice in these areas for many years.
